# Sorted Bulls’ X-Chromosome-Bearing Spermatozoa Show Increased GAPDHS Activity Correlating with Motility

**DOI:** 10.3390/genes14010235

**Published:** 2023-01-16

**Authors:** Anna A. Kapitonova, Vladimir I. Muronetz, Denis V. Pozdyshev

**Affiliations:** 1 Faculty of Biology, Lomonosov Moscow State University, 119234 Moscow, Russia; 2 A.N. Bach Institute of Biochemistry, Federal Research Center of Biotechnology of the Russian Academy of Sciences, 119071 Moscow, Russia; 3 Belozersky Institute of Physico-Chemical Biology, Lomonosov Moscow State University, 119234 Moscow, Russia; 4 Faculty of Bioengineering and Bioinformatics, Lomonosov Moscow State University, 119234 Moscow, Russia

**Keywords:** GAPDHS, sexed semen, X/Y-chromosome-bearing spermatozoa, cryopreserved semen, spermatozoa motility

## Abstract

Sperm sexing is a technique for spermatozoa sorting into populations enriched with X- or Y-chromosome-bearing cells and is widely used in the dairy industry. Investigation of the characteristics of sorted semen is of practical interest, because it could contribute to the enhancement of sexed semen fertility characteristics, which are currently lower than those of conventional semen. Comparison of a spermatozoa population enriched with X-chromosome-bearing cells to a mixed population is also intriguing in the context of potential differences that drive the mechanisms of primary sex-ratio determination. In this work, sexed (X spermatozoa) and conventional spermatozoa of Holstein bulls were analyzed for the content and enzymatic activity of GAPDHS, a sperm-specific isoform of glyceraldehyde-3-phosphate dehydrogenase that plays a significant role in the regulation of flagellar activity. No difference in the amount of this glycolysis enzyme per cell was revealed, but, notably, GAPDHS enzymatic activity in the sexed samples was significantly higher. Enzymatic activity among the group of sexed but not conventional sperm samples positively correlated with spermatozoa motility, which indicates the significant role of this enzyme for the sorted cells population.

## 1. Introduction

Predetermination of the sex of farm animals largely contributes to the herd’s reproductive efficiency. For this purpose, artificial insemination with sexed cryopreserved sperm is used for the dairy and beef industry. However, this technology has significant disadvantages—sexed bull semen shows reduced fertility characteristics in comparison with conventional sperm [1,2]. This phenomenon may be related to the sexing protocol. Prior to sperm cryopreservation, the protocol involved several steps, which may cause physical and biochemical changes affecting sperm function. These main steps are sperm dilution, treatment with DNA-binding dye Hoechst 33342 and cell sorting itself [3]. Identification of the specific biochemical alterations of sorted spermatozoa is still an unsolved problem. Of particular interest are the biochemical changes affecting sperm motility, because spermatozoa motility has been shown to correlate positively with fertility [4].

This work focuses on the characterization of glyceraldehyde-3-phosphate dehydrogenase (GAPDHS) in sexed (X spermatozoa) and conventional semen. GAPDHS is a sperm-specific isoform of the glycolysis enzyme that plays a key role in the energy metabolism of the spermatozoa [5,6]. It is also known that functional GAPDHS is required for sperm motility [7]. Interestingly, differential proteomics data indicate increased content of this enzyme in a population of male gametes enriched with X-chromosome-bearing cells in comparison with a Y-enriched population [8]. These facts make GAPDHS an interesting subject to study as a potential participant in sexed sperm motility regulation mechanisms. Today there are no data on the characteristics of this enzyme in cryopreserved sorted and unsorted bovine semen. 

In this investigation, the protein content and enzymatic activity of GAPDHS along with subjective spermatozoa motility were evaluated for sexed and conventional bovine sperm samples. Unsorted and X-chromosome-spermatozoa-enriched samples from Holstein bulls were used, as Holstein cattle represent one of the most popular cow breeds for dairy farming. Our data show that for sexed sperm samples, GAPDHS activity positively correlates with spermatozoa motility, which allows us to consider GAPDHS as a potential selection marker in dairy cattle breeding.

## 2. Materials and Methods

### 2.1. Biological Samples

The analyzed material consisted of commercially available, cryopreserved Holstein bull semen. Samples of sexed and conventional semen from 19 pedigree Holstein bulls provided by STgenetics (Navasota, TX, USA) and the “Moskovskoye” (Moscow, Russia) joint-stock company were used. For five bulls among them, sperm was supplied in two variants: non-sorted and sexed. The total performance index (TPI 2022) for analyzed bulls was in the range of +1793 to +2712.

### 2.2. Western Blot Analysis of GAPDHS Content in Samples

Rabbit polyclonal antibodies against human GAPDHS were used as the primary antibody (1:2500 dilution). Anti-rabbit goat immunoglobulins were applied as the secondary antibody (HRP goat anti-rabbit, A6154 (Merck, Darmstadt, Germany), 1:10,000 dilution).

Fifty or one hundred thousand cells in 10–20 μL were taken from each sperm sample after the first washing step and centrifugation for independent and dependent samples, respectively (in triplicate). The aliquots were stored at −20 °C when necessary. After thawing, a 4× sample buffer with ß-mercaptoethanol was added, followed by incubation at 90 °C for 10 min. SDS-PAGE was carried out with 6% stacking and 12.5% resolving gels according to Laemmli [9]. Proteins were then transferred from polyacrylamide gel to a PVDF membrane (Millipore, Burlington, MA, USA). Thermo Fisher Scientific (Waltham, MA, USA) No. 26616 protein ladder was used as the molecular weight standard.

The membrane was incubated in a 5% skimmed milk solution in TBST (tris-buffered saline, 0.05% Tween-20) for 60 min at room temperature. After triple washing with TBST for 5 min, the membrane was incubated for 90 min at room temperature with primary antibodies in TBST (the concentration of antibodies in the working solution was 2.41 µg/mL). After the next triple washing with TBST, the membrane was incubated for 60 min at room temperature with secondary antibodies, conjugated with horseradish peroxidase and diluted to 1:10,000. The membrane was then triple washed in TBST for 5 min and the signal was detected using an enhanced chemiluminescence kit (WesternBright ECL, San Jose, CA, USA) and X-ray film (Kodak MXG, NY, USA).

The amount of protein was estimated by densitometry in the ImageJ program using standard curves, based on recombinant human GAPDHS without the N-terminal region. The recombinant protein production has been described previously [10]. For comparative analysis of GAPDHS content in sexed and conventional samples, signals from the same membrane were compared.

### 2.3. GAPDHS Activity Assay

Cells after the first washing step, centrifugation and motility analysis (see Section 2.4) were twice centrifuged in 2 mL of PBS (15 min, 1500× *g*, 4 °C); the supernatant was discarded. The pellet was resuspended in activity measurement buffer (0.05 M glycine, 5 mM EDTA, 50 mM K_3_PO_4_, pH 9.0); the cell count was measured in a hemocytometer. Samples (1 million cells in 200–300 µL of activity measurement buffer with 5 mM dithiothreitol (DTT)) were then sonicated (10% amplitude, 5 s) using a Branson Digital Sonifier 450 (Marshall scientific, Hampton, NH, USA) and incubated at room temperature for 25 min.

All measurements were performed at room temperature on a UV-1800 spectrophotometer (Shimadzu, Kyoto, Japan). Activity was measured for 55 s with a kinetic interval of 0.1 s using the absorption peak at 340 nm (ε = 6220 M^−1^ × cm^−1^); the sample without glyceraldehyde 3-phosphate was used as a control. The 1 mL measurement sample contained buffer, 1 mM NAD+ (>10 KM [5]) and 1 mM glyceraldehyde 3-phosphate (≈2 KM [5]). The entire volume of the prepared sample was added. 

The data obtained in ΔA_340_/min were converted to ΔC(NAD^+^)/min. Taking into account the volume of the sample and the amount of GAPDHS (determined by Western blotting), the enzyme activity was evaluated in µmol substrate/(min·10^9^ cells) or µmol substrate/(min·mg GAPDHS).

### 2.4. Spermatozoa Motility Analysis

Straws with frozen, sorted (X spermatozoa) or conventional semen were thawed for 30 s in a water bath at 37 °C. The straw content was mixed with 10 mL of Biggers–Whitten–Whittingham (BWW) medium without lactate and pyruvate to reduce the contribution of oxidative phosphorylation to ATP production in spermatozoa (5.54 g/L NaCl, 0.356 g/L KCl, 0.294 g/L MgSO_4_·7H_2_O, 0.25 g/L CaCl_2_·2H_2_O, 0.162 g/L KH_2_PO_4_, pH 7.4, with freshly added 0.366 g/L KHCO3, 20 mM HEPES, 1 g/L d-glucose, 3.5 g/L bovine serum albumin) and centrifuged for 10 min 500× *g* at room temperature. The cell count was determined after pellet resuspension. To analyze sperm motility, 30–60 µL of suspension (2 × 10^6^ cells/mL) were incubated at 37 °C for 5 and 60 min. The microscope stage and slides were also preheated at 37 °C. The motility measurements were taken using 3 wet-mount slides (6.5 µL per slide with 18 mm × 18 mm coverslip); at least 5 random fields of view were selected and at least 70 cells were counted on each slide. The motility classification system included progressively motile, non-progressively motile (circular movement, oscillatory movements of the head or beating of the flagellum without progressive movement) and immobile spermatozoa. General motility summarized progressively motile and non-progressively motile spermatozoa counts. The sizes of the motility groups were calculated for each slide, and the final percentage was determined as the average of three slides.

### 2.5. Statistical Analysis

GraphPad Prism 9.0.2 (Boston, MA, USA) and Microsoft Excel (Redmond, WA, USA) was used for statistical analysis and visualization. All data were tested for normal distribution via the Shapiro–Wilk and Kolmogorov–Smirnov tests. If data were normally distributed, Student’s parametric *t*-test was applied for independent and dependent samples, respectively. The significance level α was set equal to 0.01 (the Shapiro–Wilk test for data in the correlation assessment) and 0.05 (for Student’s parametric *t*-test). The Pearson test was used to analyze the correlation.

## 3. Results

### 3.1. The Quantity of GAPDHS Enzyme per Cell in Conventional Semen Samples and Samples Enriched with X-Chromosome-Bearing Spermatozoa Does Not Differ

To evaluate the relative content of GAPDHS enzyme in cells from frozen-thawed samples of sexed (X spermatozoa) and conventional bovine semen, a Western blot analysis was performed with antibodies against GAPDHS, previously obtained in our laboratory [10]. These rabbit polyclonal antibodies do not have cross-reactivity with the somatic isoform of glyceraldehyde-3-phosphate dehydrogenase and provide high-sensitivity GAPDHS detection; a robust signal was obtained starting from 50 thousand spermatozoa in a sample. The small number of cells for detection in the Western blot assay made it possible to determine cell motility, GAPDHS content, and its enzymatic activity from a single straw.

**Figure 1:**
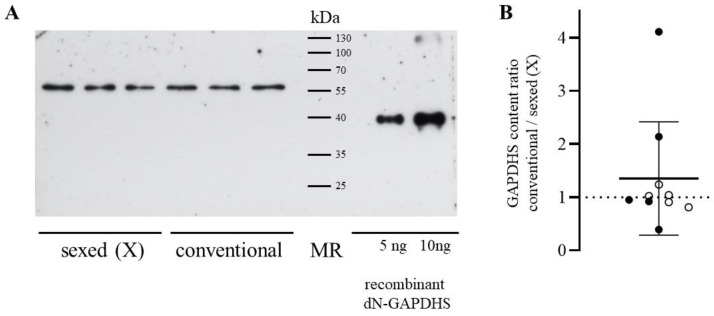
Relative GAPDHS content in the spermatozoa from sexed and conventional bull semen samples (**A**). Western blot of sperm samples derived from two bulls with antibodies against GAPDHS. Recombinant GAPDHS with no N-domain and with known concentrations was used as a control for quantitative analysis. For each sample, 50,000 cells per track were loaded in three repetitions. (**B**). The ratio of GAPDHS content in unsorted semen to GAPDHS content in sexed semen evaluated by densitometry for samples derived from the same bull (white circles) or from different animals (shown with black dots). The bold line corresponds to the mean value and error bars indicate standard deviation.

Due to the abundance of proline residues, the GAPDHS molecule yields a band at about 56 kDa when electrophoresed in a polyacrylamide gel [11]. Human sperm lysates can yield an additional band at around 37 kDa, which probably corresponds to proteolyzed GAPDHS [10]. In the case of bovine spermatozoa, no additional bands were observed; the calculation of the amount of protein was performed using the main band. Different amounts of recombinant GAPDHS without the N-terminal domain were used for calibration. The results of quantitative assessment show that the content of GAPDHS in the samples averaged 6.89 ± 0.72 ng per 100 thousand cells. The amount of this enzyme in the sexed and conventional semen samples did not differ (*p*-value = 0.31, unpaired two-tailed *t*-test, N = 20 samples). As the content of GAPDHS in spermatozoa varies for different bulls, we compared the amount of this protein in unsorted and sorted spermatozoa derived from the same animal (Figure 1). This approach also revealed no statistically significant differences (*p*-value = 0.96, paired two-tailed *t*-test, N = 10 samples).

Cryopreservation and sorting are associated with significant cell stress and can lead to irreversible inhibition and denaturation of the enzymes. As the amount of GAPDHS is equal in bovine spermatozoa from an unsorted population and an X-chromosome-bearing population, it was important to evaluate the activity of this enzyme.

### 3.2. GAPDHS Enzymatic Activity in Conventional Semen Samples Is Lower Than in Samples Enriched with X-Chromosome-Bearing Spermatozoa

**Figure 2:**
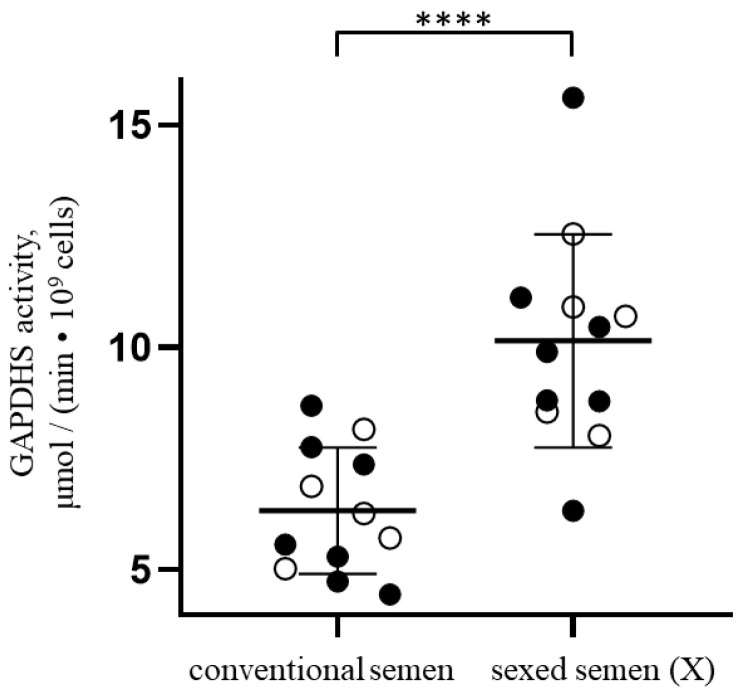
GAPDHS enzymatic activity in spermatozoa from frozen-thawed samples of sexed (X spermatozoa) and conventional bovine sperm derived from the same bull (white circles) or from different animals (shown with black dots). **** *p*-value < 0.0001. The bold line corresponds to the mean value and error bars indicate standard deviation.

As Western blot analysis does not provide information on the functional characteristics of GAPDHS, the next stage was to measure activity of this enzyme for the frozen-thawed samples of sexed and unsorted bovine sperm. Sample preparation for the enzymatic assay was performed in parallel with the measurement of the GAPDHS amount described above immediately after thawing the sample. It is important to mention that the preparation protocol includes a spermatozoa washing step to remove the storage medium and a step of reduction of cysteine groups with DTT. The GAPDHS activity in the sexed samples was significantly higher than in samples of conventional semen according to the results of measurements—10.15 ± 2.39 µmol/(min·10^9^ cells) for the sorted vs. 6.33 ± 1.42 µmol/(min·10^9^ cells) for conventional semen (Figure 2). Normalized to protein content, the enzymatic activity for sorted spermatozoa is also significantly higher than for the mixed population (143 ± 31 µmol/(min·mg GAPDHS) for the sexed vs. 96 ± 27 µmol/(min·mg GAPDHS) for unsorted semen). 

GAPDHS is tightly bound to the spermatozoa fibrous sheath and has a structural and energetic function; therefore, the next step was to analyze the spermatozoa motility of the samples.

### 3.3. GAPDHS Enzymatic Activity in Sexed Semen Samples Positively Correlates with Spermatozoa Motility

**Figure 3:**
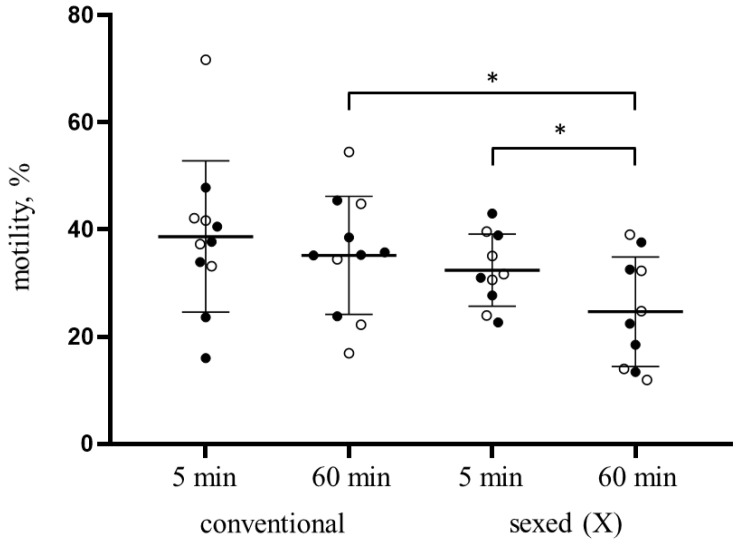
Percentage of motile sperm among the total number of gametes at the beginning (5 min post-thawing) and at the end (60 min post-thawing) of incubation at 37 °C in frozen-thawed samples of sexed (X spermatozoa) and conventional bovine sperm derived from the same bull (white circles) or from different animals (shown with black dots). * *p*-value < 0.05. The bold line corresponds to mean value and error bars indicate standard deviation.

GAPDHS is essential for normal sperm motility [7]. At the same time, sperm motility is an important characteristic of sperm quality, associated with fertilizing potential. The proportion of motile spermatozoa for frozen-thawed bovine semen samples was determined after thawing and incubation in a special buffer for 5 and 60 min at 37 °C. In the case of conventional sperm samples, the percentage of motile sperm in relation to the overall cell number at the beginning of incubation ranged from 16 to 71.7%, while for sexed samples (X spermatozoa) the variation was a little less—from 17.3 to 51.1%. Statistical analysis of the results revealed no significant difference between the general cell motility in sorted and unsorted samples—the values for all samples were 32.43 ± 6.73% and 38.70 ± 14.12%, respectively. The difference in mobility is insignificant both for paired and independent samples (sperm derived from the same bull or from different animals) (Figure 3).

After incubation in an incomplete BWW medium for one hour after thawing, the average sperm motility values in the samples of the sexed and conventional sperm decreased and were 24.68 ± 10.22% and 35.18 ± 11.01%, respectively. The difference in the motility of sorted and unsorted spermatozoa after incubation was statistically significant. Notably, although the decrease in motility for conventional sperm at the end of incubation was insignificant, crucially more immobile cells were observed in sexed samples in comparison with start point.

**Figure 4:**
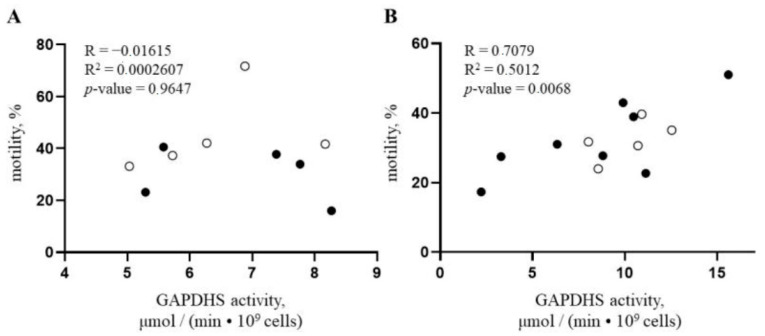
Correlation between GAPDHS activity and the percentage of motile spermatozoa five minutes post-thawing for samples of conventional bovine sperm (**A**) and for samples enriched with X-chromosome-bearing gametes (**B**). Analyzed frozen-thawed samples derived from the same bull are shown with white circles and samples derived from different animals are shown with black dots. Statistics were calculated using the Pearson correlation coefficient.

As cell motility data were obtained for the samples with measured GAPDHS activity levels, a correlation analysis was performed for these parameters. No correlation between motility and enzymatic activity after incubation for 60 min after thawing for both types of samples was observed. The percentage of motile spermatozoa after incubation for 5 min post-thawing correlated with the GAPDHS activity values in the respective samples only in the case of sexed samples, but not for conventional semen samples (Figure 4). Thus, GAPDHS activity is not a key factor in determining sperm motility in frozen-thawed conventional sperm samples but becomes essential in the sorted X-chromosome-bearing cell population.

## 4. Discussion

Artificial insemination combined with sperm cryopreservation technology is currently the main method in dairy cattle breeding in developed countries [12]. Prior to cryopreservation, sperm can be divided into fractions of X- or Y-chromosome-bearing spermatozoa, allowing for the production of offspring of the preferred sex. The most-studied method of sperm sexing is fluorescence-activated cell sorting. This method is based on the difference in DNA content between X and Y chromosomes detected by staining with Hoechst 33342 dye. This complex sorting process is followed by the cryopreservation stage, in which sperm are also exposed to stressful conditions, such as temperature changes and osmotic and toxic stress caused by cryoprotectants [13]. An analysis of data accumulated over more than 20 years shows that the fertility rate of sexed bovine semen is significantly reduced compared to unsorted [14]. The precise reasons for this phenomenon are the subject of research. Previously, a comparison of frozen-thawed sex sorted (X spermatozoa) and frozen-thawed unsorted bovine semen showed changes in kinetic parameters, viability, mitochondrial potential and acrosome integrity; there was a difference in the content of proteins participating in capacitation and sperm–egg fusion [15]. A difference in the content of some enzymes involved in the cell’s energy metabolism was also found [16]. The reported work focuses on the molecular features of glycolysis enzyme GAPDHS in sorted and conventional semen samples. We showed no difference in the amount of this protein per cell for such semen samples using Western blot analysis; this corresponds to the results of differential proteomics published earlier. 

The analyzed sexed semen samples contain at least 90% X-chromosome-bearing spermatozoa [17], while the percentage of such cells in conventional semen samples is about 50% [18]. The opportunity to isolate populations of male gametes with X and Y chromosomes was previously used in a number of studies on differential transcriptomics [19,20] and proteomics [8,21,22] of these groups of cells. One of these studies reported that GAPDHS was detected in higher amounts in spermatozoa bearing an X chromosome [8]. However, our comparison of sorted and mixed populations does not support this data by Western blot analysis.

A significant increase in GAPDHS activity was found in the bovine sexed samples in comparison with conventional sperm, while this enzyme content for both groups was equal. The mean values for GAPDHS-specific activity are comparable to those for other mammalian species. For example, for unsorted sperm they are nearly twice as high as the values for the human ortholog (the activity is about 50 units/mg for human [5] vs. 96 units/mg for bull) and slightly higher than boar GAPDHS (the activity is about 4–5 units/(10^9^ cells) for boar [23] vs. 6.33 units/(10^9^ cells) for bull).

There are several explanations for the significant difference in GAPDHS activity in sexed and unsorted samples. First, it could be the result of differences in storage buffers, e.g., antioxidant additives. Previously increased levels of reactive oxygen species for sorted bovine semen were shown [24] and changes were made to the buffer formulations, accordingly [25]. In our enzymatic assay, the spermatozoa were washed from the initial medium, and the sample was incubated with DTT, since GAPDHS has a cysteine residue in the active center, which is extremely sensitive to oxidative stress [26]. However, these procedures could not avoid irreversible inhibition or denaturation of the enzyme during sample storage prior to the enzymatic test.

Secondly, the increased level of GAPDHS activity in X spermatozoa may be related to peculiarities in activity regulation in the sorted population. In addition to changes in the ratio of X- or Y-chromosome-bearing cells, the unsorted sample probably contains cells of varying developmental stages and conditions. Sperm sorting could eliminate defective cells with a less active enzyme, for instance. Conversely, the obtained result could be associated with an increased percentage of capacitated spermatozoa, since it is known that the membrane state of the sexed cells is more associated with the in vitro capacitation process [15]. Finally, the difference shown in GAPDHS activity could be explained directly by the specific character of enzyme regulation in X and Y spermatozoa.

A correlation analysis of GAPDHS activity and subjective sperm motility was performed. The motility values after 5 min of incubation in incomplete BWW medium correlated with the enzyme activity only in the group of sexed sperm samples. This observation is particularly important because motility is a widely used characteristic of semen quality, and subjective spermatozoa motility has been shown to correlate positively with fertility [4]. Thus, for sexed semen, GAPDHS activity can be a fertility indicator that provides additional information for the evaluation of semen batch quality or even for the selection of bulls in cattle breeding. The latter is especially important considering that the fertility of bull sires is a complex trait with relatively low heritability. 

GAPDHS activity correlation with sexed spermatozoa motility is probably related to the fact that the sorting procedure selects a more state-homogeneous group of cells. Cell motility is regulated by many participants, and mechanical flagellar bending is provided by phosphorylation of the outer dynein arms of the axoneme, which leads to sliding of peripheral microtubule doublets [27]. Motion energy can be provided by glycolysis, the pentose phosphate pathway and oxidative phosphorylation. Thus, GAPDHS is only one of the factors affecting cell motility. In addition, it was shown to become determinant only for the selected sperm population. 

The obtained data support the involvement of GAPDHS in cell motility mechanisms. Previously, it was shown that the activity of this enzyme correlated with the motility of sperm in conventional stallion and human semen—GAPDHS activity was higher in a more motile group of spermatozoa [28]. Furthermore, the GAPDHS content in the sperm of men with asthenozoospermia is lower than in the sperm of the control group [29]. The aforementioned studies on the comparison of X and Y bovine spermatozoa transcriptomes and proteomes agreed with the conclusion that the key differences mainly concern the flagellar structural proteins and proteins of energy metabolism. We propose that our findings on the positive correlation between GAPDHS activity and motility of X-chromosome-bearing spermatozoa may complete this picture and bring us closer to understanding the mechanisms of sex predetermination.

## 5. Conclusions

Frozen-thawed sexed sperm samples (X-chromosome-bearing cells) were compared with frozen-thawed unsorted Holstein bull semen samples regarding three parameters—GAPDHS content, GAPDHS enzymatic activity and spermatozoa motility. While the amount of the enzyme and cell motility in the samples were statistically indistinguishable, a crucial increase in GAPDHS activity was shown in the sexed samples. The significant positive correlation between GAPDHS enzymatic activity and spermatozoa motility for semen samples enriched with X-chromosome-bearing spermatozoa was also identified, which allows us to consider GAPDHS as a potential selection marker in dairy cattle breeding.

## Data Availability

The data supporting reported results can be requested from the corresponding author.
